# One-Step Hydrothermal Synthesis of P25 @ Few Layered MoS_2_ Nanosheets toward Enhanced Bi-catalytic Activities: Photocatalysis and Electrocatalysis

**DOI:** 10.3390/nano9111636

**Published:** 2019-11-18

**Authors:** Fang Zhou, Zhiguang Zhang, Zhihua Wang, Yajing Wang, Liping Xu, Qiang Wang, Wenjun Liu

**Affiliations:** 1 School of Physics, Harbin Institute of Technology, Harbin 150001, China; 15B911003@hit.edu.cn; 2 College of Physical Science & Technology, Yangzhou University, Yangzhou 225002, China; 3 College of Science & Information, Qingdao Agricultural University, Qingdao 266109, China; zhangzhiguang@qau.edu.cn (Z.Z.); misszhihua@qau.edu.cn (Z.W.); yajingphysics@qau.edu.cn (Y.W.); xlpzzd@qau.edu.cn (L.X.)

**Keywords:** P25, MoS_2_, bi-catalysis, photocatalysis, electrocatlysis

## Abstract

P25 loaded few layered molybdenum disulfide (MoS_2_) nanosheets (P25@MoS_2_) are successfully synthesized through a facile one-step hydrothermal process. The bi-catalytic activities, i.e., photocatalytic and electrocatalytic activities, of the as-prepared nanomaterials have been investigated. For the as-prepared products, the photocatalytic performances were investigated by degrading simulated pollutant under sunlight irradiation, and the hydrogen evolution reaction evaluated the electrocatalytic performances. The results indicate that P25@MoS_2_ possesses excellent activities in both photocatalysis and electrocatalysis. The presence of MoS_2_ broadens the light absorption range of P25 and improves the separation and transformation efficiency of photogenerated carriers, thus improving its photocatalytic performance. The existence of P25 inhibits the aggregation of MoS_2_ to form more dispersed MoS_2_ nanosheets with only few layers increasing its active sites. Thereby, the electrocatalytic performance is heightened. The excellent multifunction makes the as-prepared P25@MoS_2_ a promising material in the fields of environment and energy.

## 1. Introduction

In recent years, the environmental pollution and energy shortage that have been caused by the extensive use of fossil energy threatened the healthy and survival of human beings. Therefore, the bottleneck problem of energy and environmental pollution, which restricts human development, needs to be urgently solved. More renewable energy and less fossil energy are advantageous for environment and sustainable development. Among many renewable resources, solar energy has become the preferred alternative energy due to its advantages of time-durability and environmental friendliness.

Among the ways of utilizing solar energy, photocatalytic technology is more popular due to of its direct use of clean solar energy to solve environmental pollution problems. Many semiconductor nanomaterials, exhibiting an excellent performance of photocatalysis, have been investigated, such as ZnO, CuO, CdSe, etc. [1,2,3,4]. Among these semiconductors, titanium dioxide (TiO_2_) is undoubtedly the most shining one. Although TiO_2_ has extra-outstanding activity in photocatalysis, it still cannot get rid of the two inherent weaknesses: wide-band-gap and low quantum efficiency [5]. The combination of two semiconductor materials with a matched energy level is one effective way to solve these two deficiencies of TiO_2_ [6,7].

In addition, using solar energy to catalyze the production of hydrogen is intensively concerned at present [8,9,10]. However, the direct use of solar energy for photocatalytic hydrogen production will be strongly affected by weather, and thus affect the stability and sustainability of hydrogen production. Electrocatalytic hydrogen production is a promising means of preparing alternative energy sources, owing to the fact that various renewable energies (wind, waves, tide, etc.) can be conversed to electric energy [11]. In this way, various renewable energy can be conversed, stored, and transported by using hydrogen as the medium [12]. Many nanomaterials for electrocatalysis have been produced and researched, such as Ni_2_P [13], CoSe_2_ [14], C_3_N_4_@NG [15], molybdenum disulfide (MoS_2_) [16], and so on.

Potocatalysis and electrocatalysis, both effective ways, have their own advantages in utilizing renewable energies. Therefore, designing a system that can operate under light and electricity is meaningful. The core of this system is to synthesize a nanomaterial possessing both excellent photocatalysis and excellent electrocatalysis. For this purpose, the composite of commercial P25 and few layered MoS_2_ nanosheets (P25@MoS_2_) is synthesized.

P25 is consists of TiO_2_ nanoparticles with an average size of 25 nanometers, which has extra outstanding activity in photocatalysis, and it has been considered as a reference of photocatalyst [17]. However, P25 has no excellent electrocatalytic performances. MoS_2_ has excellent electrocatalytic activity, especially in hydrogen evolution [18,19]. It has been reported that MoS_2_ edge structure resemble nitrogenase active site, and the free energy of MoS_2_ edge is close to Pt [20]. However, MoS_2_ has no excellent photocatalytic performance. When considering that P25 has excellent photocatalysis and MoS_2_ has excellent electrocatalysis, the combination of P25 and MoS_2_ is expected to possess excellent bicatalysis- photocatalysis and electrocatalysis.

Zheng et al. synthesized a hierarchical MoS_2_ nanosheet@TiO_2_ nanotube array by combining the anodization method with the hydrothermal method for enhanced photocatalysis in degrading rhodamine B (RhB) [21]. Song and co-workers prepared the MoS_2_/TiO_2_ hybrids with various interfaces by a three-step hydrothermal method for electrocatalytic hydrogen evolution reaction (HER) [22]. The MoS_2_/TiO_2_ etching in H_2_SO_4_ had the best performance in all of the samples. Ma et al. reported flower-like MoS_2_@TiO_2_ nanohybrids for hydrogen evolution by the two-step hydrothermal method [23]. The results showed that MoS_2_@TiO_2_ had improved photocatalysis, and the sample (14.6 wt% MoS_2_) had an onset overpotential of −340 mV (vs RHE) and a Tafel slope of 81 mV dec^−1^. However, these excellent achievements need a complex process to composite MoS_2_@TiO_2_. Moreover, the electrocatalytic properties of some samples that are mentioned above are not good enough. Therefore, synthesizing MoS_2_@TiO_2_ by a simple method and keeping excellent bi-catalytic activity are meaningful.

In this work, P25@MoS_2_ is synthesized by a simple one-step hydrothermal method. Photodegrading RhB and HER are selected for evaluating the performances of P25@MoS_2_ in photocatalysis and electrocatalysis, respectively. P25@MoS_2_ greatly enhances the degrading efficiency due to matched energy levels of P25 and MoS_2_. Owing to the fact that P25 can hamper the self-assembling of MoS_2_, more active sites in MoS_2_ are exposed to improve the HER efficiency. It has been confirmed that both the photocatalytic and electrocatalytic performance of the as-prepared products have been improved, which makes the P25@MoS_2_ a promising material in the fields of environment and energy.

## 2. Experimental

### 2.1. Chemical Reagents

Degussa P25 (80% anatase and 20% rutile) was purchased from Evonik Degussa Company (Shanghai, China). Sodium molybdate (NaMoO_4_·2H_2_O, AR, 99.0%), hydroxylamine hydrochloride (NH_2_OH·HCl, AR, 98.5%), and thiourea (CH_4_N_2_S, AR, 99.0%) were purchased from Sinopharm Chemical Reagent Co., Ltd (Shanghai, China). All of the chemicals were used without any further purification, and all of the solutions were prepared with deionized water.

### 2.2. Synthesis of P25@MoS_2_

The synthesizition process of P25@MoS_2_ was described, as follows: typically, 90 mg NaMoO_4_·2H_2_O, 142 mg CH_4_N_2_S, and 52 mg NH_2_OH·HCl were dissolved in 35 mL deionized water, followed by stirring until clear and homogeneous. The above solution was transferred to a 50 mL Teflon lining-lined stainless steel autoclave. P25 powder was immersed in the solution by stirring and ultrasonic dispersing. Afterwards, the suspension was heated in an electric oven at 200 °C for 24 h. When naturally cooling down to room temperature, the final product was centrifuged and washed several times with deionized water and absolute ethanol, and then dried at 60 °C for 24 h. By adjusting the P25 content, the P25@MoS_2_ with mass ratios of 2:1, 1:1, and 1:2 were synthesized, and donated as PM21, PM11, and PM12, respectively. Pure MoS_2_ was synthesized with the same process without P25.

### 2.3. Characterizations

X-ray diffraction (XRD, Shimadzu7000, Kyoto, Japan) characterization was applied to investigate the crystal phases of the as-synthesized samples. Field emission scanning electron microscopy (FESEM, S-4800, Hitachi, Tokyo, Japan) and high resolution transmission electron microscope (HRTEM, Tecnai G2 F30 S-TWIN, FEI, Hillsboro, OR, USA) were employed to study the morphologies and microscopic structures. X-ray photoelectron spectra (XPS, ESCALAB250Xi, ThermoFisher Scientific, Waltham, MA, USA) were utilized to analyze the chemical components and states. The UV-Vis diffuse reflection spectra (DRS) were recorded by a Shimadzu UV-2600 UV-visible spectrophotometer (Kyoto, Japan). The efficiency of electron–hole pair separation was verified by the photoluminescence spectrophotometer (PL, F-2500, Hitachi, Tokyo, Japan), with an excitation wavelength at 320 nm. The electrochemical workstation (CHI 660E, Chenhua, Shanghai, China) was used to test the activities of hydrogen production.

### 2.4. Photodegradation Studies

For comparison, the samples of P25, MoS_2_, PM21, PM11, and PM12 were employed in the photodegradation experiments. 5 mg photocatalysts are suspended in 50 mL RhB solution (15 mg L^−1^), and then the suspends were stirred in the dark for 30 min. to ensure the adsorption-desorption equilibrium. After that, the suspensions were exposed to a 350W Xe lamp (Figure S1) for 120 min. Recycling experiments were carried out while using PM11 for five times under the same condition. Before each recycling, PM11 was washed, centrifuged several times, and then dried for 12 h at 80 ℃.

### 2.5. Hydrogen Production Studies

The samples as the working electrode are prepared, as following: the prepared powder (4.8 mg), acetylene black (1.2 mg), ethanol solution (300 µL), Nafion (5 wt %, 30 µL), and deionized water (300 µL) are added together and ultrasonically treated for 30 min. The electrochemical polarization curves are investigated in a typical three-electrode setup: Ag/AgCl electrode as the reference electrode, the prepared samples on a glassy carbon electrode as the working electrode and the Pt foil (15 mm× 15 mm) as the counter electrode. The experiments are carried out in 0.5M H_2_SO_4_ solution.

## 3. Results and Discussions

### 3.1. Structure and Morphology Characteristics

**Figure 1:**
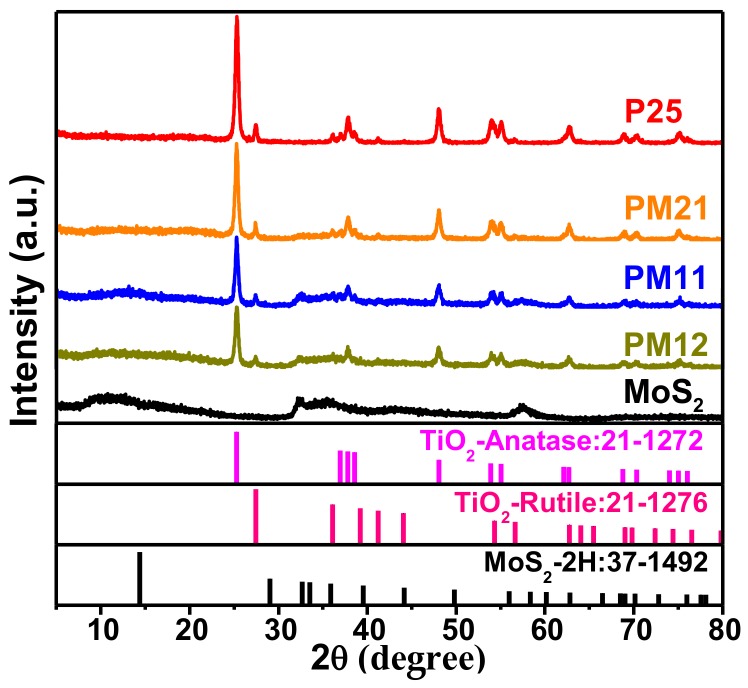
X-ray diffraction (XRD) patterns of pure P25, PM21, PM11, PM12, and molybdenum disulfide (MoS_2_).

Figure 1 shows the XRD patterns of the as-prepared samples. For pure P25, the detected peaks can be assigned to anatase phase TiO_2_ (JCPDS card No. 21-1272) and rutile phase TiO_2_ (JCPDS card No. 21-1276). For pure MoS_2_, all diffraction peaks matched with hexagonal phase MoS_2_ (JCPDS card No. 37-1492) very well, except for the diffraction peak of (002) plane. The decreased diffraction angle of (002) plane indicated the increased interplanar distance, which could be ascribed as the ions or particles insertion between layers [24,25]. In Figure 1, with the content of P25 decreases, the intensities of P25 diffraction peaks gradually decreases, implying that the amount of MoS_2_ increases gradually. Similarly, with the content of MoS_2_ increases, the diffraction peaks of MoS_2_ become more and more similar to pure MoS_2_. Interestingly, the diffraction peak of (002) plane of PM11 (13°) seems more noticeable and closer to standard value (14.4°) than that of PM12 (11°).

**Figure 2:**
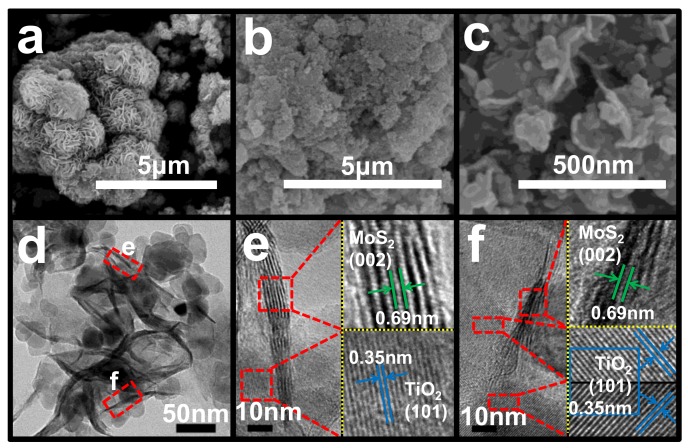
(**a**) Field emission scanning electron microscopy (FESEM) image of pure MoS_2_. (**b**) Low and (**c**) high magnification FESEM images of PM11 respectively. (**d**) Transmission electron microscope (TEM) image of PM11. (**e**,**f**) High resolution transmission electron microscope (HRTEM) images of PM11 corresponding to the selected areas in (d).

To further confirm the structure and morphology of P25@MoS_2_, FESEM, TEM, and HRTEM characterizations were employed. The FESEM images of pure MoS_2_ were obtained, as shown in Figure 2a. It is obviously that thin MoS_2_ nanosheets are self-assembled to form flower-like microspheres. Figure 2b,c depict petal-like structure of PM11, which is apparently different from pure MoS_2_. This difference should be attributed to the presence of TiO_2_, which can be further confirmed by the TEM image (Figure 2d) and HRTEM images (Figure 2e,f). Figure 2d presents a typical TEM image of PM11. It evidently displays the compositing of P25 nanoparticles and MoS_2_ nanosheets. Figure 2e,f are the corresponding HRTEM images for the selected two parts in Figure 2d, respectively. As depicted in Figure 2e,f, the lattice fringes of 0.35 nm can be assigned to the (101) plane of anatase TiO_2_ [21,26]. Besides, the interlayer spacing of 0.69 nm can be indexed to the (002) plane of 2H-MoS_2_, which is consistent with the above XRD results (the peak with diffraction angle of 13°) [24]. The TEM and HRTEM results further confirmed the formation of the heterostructure between P25 and MoS_2_.

**Figure 3:**
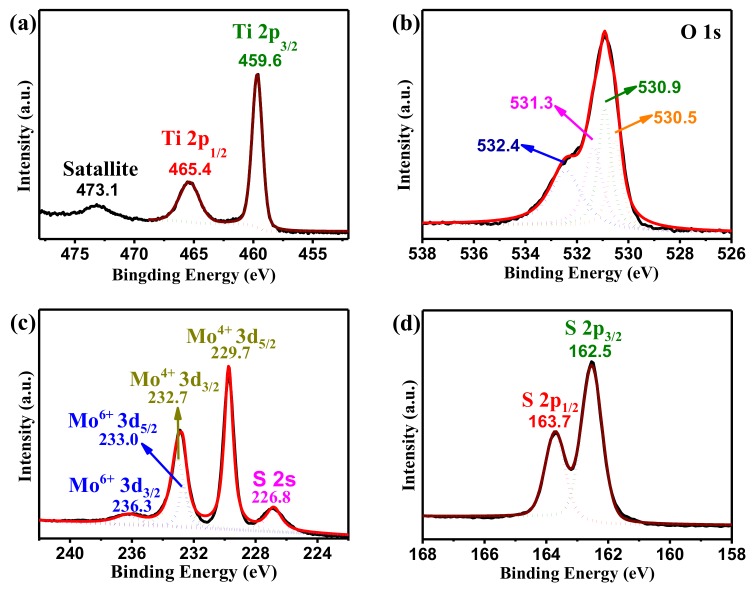
High-resolution X-ray photoelectron spectra (XPS) spectra of PM11: (**a**) Ti 2p, (**b**) O 1s, (**c**) Mo 3d, and (**d**) S 2p.

The XPS analysis was employed to investigate the chemical composition and bonding configuration of the as-prepared samples. The full XPS survey of PM11 (Figure S2) reveals the existence of Ti, O, Mo, and S, and the observed peak at 285 eV of C 1s originated from the signal of carbon in the instrument [21,23]. Figure 3 displays high-solution spectra. The two peaks located at 465.4 eV and 459.6 eV can be assigned to Ti 2p_1/2_ and Ti 2p_3/2_, respectively, as depicted in Figure 3a. The additional peak located at 473.0 eV, which shifts about 13 eV relative to Ti 2p_3/2_, is considered as a shake-up satellite [27,28]. The O 1s XPS spectrum (shown in Figure 3b) shows four peaks that correspond to four different states of O elements. The peak with the binding energy of 532.4 eV is attributed to adsorbed water (H_2_O), and the peak with the binding energy of 531.3 eV implies the formation of hydroxyl group (–OH) [29,30]. The peak at around 530.9 eV indicates the formation of Ti–O–Mo bonds between TiO_2_ and MoS_2_, and the peak at 530.5 eV is associated with Ti–O bond state of TiO_2_ [30]. The peak at 226.5 eV in Figure 3c is associated with S 2s, and the other four peaks are attributed to Mo 3d [31,32]. The two strong peaks at 232.7 eV and 229.7 eV correspond to Mo^4+^ 3d_3/2_ and Mo^4+^ 3d_5/2_, respectively [31,32]. The two weak peaks at 236.3 eV and 233.0 eV correspond to Mo^6+^ 3d_3/2_ and Mo^6+^ 3d_5/2_, respectively [31,32]. It has been reported that the appearance of Mo^6+^ implies the complete reduction reaction or oxidization of Mo^4+^ on the surface in air [31,32]. As depicted in Figure 3d, S 2p_1/2_ and S 2p_3/2_ are located at 163.7 eV and 162.5 eV, respectively [21,23]. The XPS results further confirm that MoS_2_ and P25 combine together very well.

### 3.2. Optical Properties

**Figure 4:**
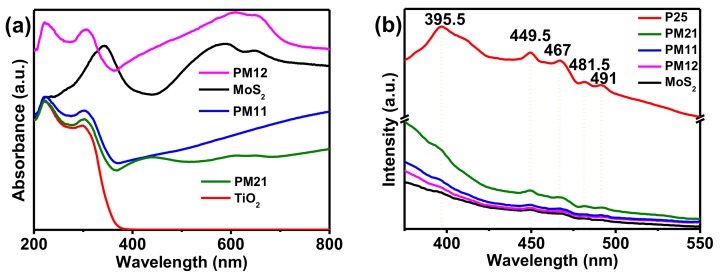
(**a**) UV-Vis diffuse reflectance spectra and (**b**) PL spectra of P25, MoS_2_, PM21, PM11, and PM12.

Figure 4a shows UV-Vis diffuse reflectance spectra. It is obvious that P25 has rather weak absorption in visible range and very high absorption in ultraviolet range. However, MoS_2_ and all of the composites (PM21, PM11, PM12) have very high absorption ability in the range of 200–800 nm. Therefore, benefitting from the loading of MoS_2_, the utilization of solar spectra for P25 has been broadened.

For the semiconductor, the characteristic of charge trapping and recombination of photogenerated electron-hole pairs can be investigated by PL emission spectrum. As exhibited in Figure 4b, the PL curve of P25 has five main emission peaks, which are located at 395.5 nm (3.14 eV), 449.5 nm (2.76 eV), 467 nm (2.66 eV), 481.5 nm (2.58 eV), and 491 nm (2.53 eV), respectively. The peak of 395.5 nm is attributed to electronic transition from the bottom of TiO_2_’s conduction band (CB) to the top of TiO_2_’s valence band (VB), which implies the recombination of photogenerated electron-hole pairs [21,33]. Additionally, the other four peaks may be associated with the emission of oxygen vacancy formed during the synthesis process [21,33]. As can be seen from Figure 4b, these five peaks that are mentioned above still exist in the samples after loading MoS_2_, meaning the presence of P25. However, the intensity of these five peaks is greatly reduced, which implies that the loading of MoS_2_ inhibits the recombination of photogenerated electron-hole pairs in P25. The optical performances of the resultant products have confirmed that the compositing between P25 and MoS_2_ can improve the absorption of solar light, and suppress the carrier recombination to heighten the separation efficiency of photogenerated electron-hole pairs.

### 3.3. Photocatalytic Performances

**Figure 5:**
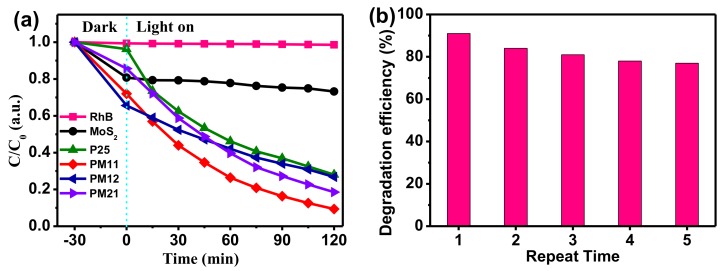
(**a**) The curves of C/C_0_ vs. time for photodegrading rhodamine B (RhB). (**b**) The degradation efficiency in recycling of PM11.

Figure 5 shows the photocatalytic performances for degrading RhB. Without photocatalysts, pure RhB exhibits no appreciable degradation, which indicates the stability of RhB under solar light irradiation. Additionally, pure MoS_2_ has a negligible degradation performance. As we know, P25 displays outstanding photodegrading activity, which is usually used as a reference for photocatalytic activity. For the three composites (PM11, PM12, and PM21), they all have higher degradation efficiencies than P25; especially, PM11 has the best photocatalytic performance, which further confirms that the composite of P25 and MoS_2_ enhances the photocatalytic performance indeed. In addition, the recycling stability is a very essential ability of photocatalyst for practical application. Figure 5b shows the recycling results of PM11 to degrade RhB. As can be seen in Figure 5b, the PM11 exhibits good stability during five continuous cycles.

The possible mechanisms (Figure S3) are discussed in the following. When the energy of photon is greater than the band gap (Eg) of semiconductor, electron (e^−^) in the valence band (VB) can be excited to the conduction band (CB) by absorbing photon. Subsequently, hole (h^+^) is left in VB, and a photo-generated electron-hole pair is produced. The h^+^ can oxidize H_2_O to create OH• species. Meanwhile, e^−^ can react with O_2_ (dissolved in solution) to make O_2_^−^• species. Afterwards, the active species for degradation are produced. By loading MoS_2_ on P25, the absorption range of P25 is broaden, which can be confirmed by the UV-Vis spectra. Therefore, more photon is absorbed to produce more species, hence enhancing the photocatalytic performance of the products. However, electron-hole pairs of TiO_2_ and MoS_2_ both have high rates of recombination, which inhibits the production of active species to lower the degrading efficiency [34,35]. Attributed to the matched energy levels, the e^−^ in CB can be transmitted from MoS_2_ to P25 and the h^+^ in VB can be effectively transmitted from P25 to MoS_2_ [21]. Therefore, the recombination of e^-^ and h^+^ is suppressed, and the intensity of the PL spectra is reduced, which is consist with the PL spectra. P25@MoS_2_ can produce much more active species due to the separation of the electron-hole pairs rapidly. P25@MoS_2_ is a photocatalyst with promising application prospect, owing to the excellent degradation performance and recycling stability.

### 3.4. Electrocatalytic Performance

**Figure 6:**
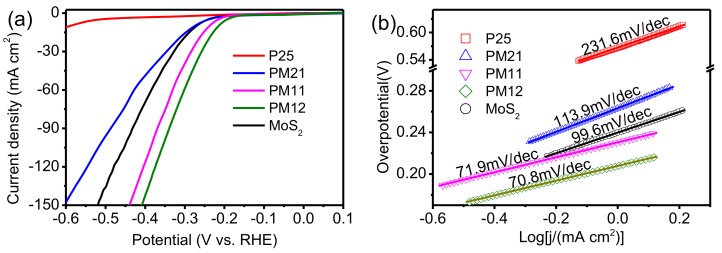
(**a**) Polarization curves and (**b**) corresponding Tafel plots.

The electrocatalytic activity of the prepared samples are studied by HER. Figure 6a shows the comparative polarization curves of those prepared samples. The overpotentials to achieve a current density of 10 mA/cm^2^ are about 590 mV (P25), 250 mV (MoS_2_), 275 mV (PM21), 237 mV (PM11), and 215 mV (PM12), respectively. It is clearly shown that P25 needs much higher overpotential than MoS_2_. This implies that the electrocatalytic performance of P25 is much lower than that of pure MoS_2_. The overpotential of PM21 is a little higher that of MoS_2_. PM11 and PM12 have the approximate overpotential and current density changes, which indicates that the electroatalytic activity of PM11 and PM12 are close.

Figure 6b shows the Tafel slope in linear portions are 231.6, 99.6, 113.9, 71.9, and 70.8 mV per decade for P25, MoS_2_, PM21, PM11, and PM12, respectively. The Tafel slope of P25 is much higher than that of MoS_2_, confirming that the electrocatalytic performance of pure MoS_2_ is much higher than that of P25. The Tafel value of PM21 is much lower than that of P25 and higher than that of MoS_2_. Additionally, PM11 and PM12 hold similar Tafel slopes and are lower than all other simples. All of the results display that MoS_2_ that is composited with P25 with an appropriate proportion can improve the performance of HER.

It has been proved that the active sites of MoS_2_ only locate at the edges but not basal plane [20]. For pure MoS_2_, it grows freely in solution (without P25) to form larger aggregated cluster with less edges. However, in the solution with P25, the MoS_2_ grows on P25 in the form of scattered nanosheets, improving the amount of edges, thereby the active sites. Therefore, the HER performance is enhanced. In composites, more P25 that is associated with less MoS_2_ means less aggregated MoS_2_, which is advantageous for HER. However, it also means that less MoS_2_ provides less active sites, which is disadvantageous for HER. More MoS_2_ is related with MoS_2_ aggregated seriously, leading to reduced active sites. Hence, the content balance between P25 and MoS_2_ should be appropriate. Among the three composites, PM21 containing the least MoS_2_ should be responsible for the lowest HER. However, PM12 with the most content of MoS_2_ in composites has little HER improvement than PM11, which should be attributed to more aggregated MoS_2_ in PM12 than in PM11.

## 4. Conclusions

Through a facile one-step hydrothermal process, P25 loaded few layered MoS_2_ nanosheets were successfully synthesized. Photocatlysis and electocatlysis performances of the as-prepared products were both investigated. Under simulated sunlight irradiation, P25@MoS_2_ exhibits enhanced photocatalytic performance. Improved HER performance had displayed that P25@MoS_2_ had excellent electrocatalytic activity. All of the results have depicted that PM11 possesses excellent bi-catalytic activities. The possible mechanisms of enhanced bi-catalytic activities for PM11 had been discussed. The effective separation and transformation of photogenerated electrons and holes should charge for the promoted performance of photocatalysis. Additionally, the structure of scattered nanosheets of MoS_2_ with more active sites might be responsible for the enhanced HER performance. The excellent multifunction makes PM11 a promising candidate in environmental protection, as well as in energy conversion, storage, and transport.

## Supplementary Materials

The following are available online at https://www.mdpi.com/2079-4991/9/11/1636/s1, Figure S1: The schematic diagram of the photocatalytic system: (1) and (2) cooling water entrance, (3) and (4) cooling water outlet, (5) quartz well, (6) Xe lamp, (7) quartz reactor, (8) magnetic stirrer. The distance between the quartz reactor and lamp is about 12 cm, and the optical density of light source is about 120 mW/cm2, Figure S2: The full scan XPS spectrum of the PM11, Figure S3: Schematic illustration of the mechanisms in degrading process.
