# Supplementary material for: One-Step Hydrothermal Synthesis of P25 @ Few Layered MoS2 Nanosheets toward Enhanced Bi-catalytic Activities: Photocatalysis and Electrocatalysis
Source: Nanomaterials (Basel). 2019 Nov 18;9(11):1636. doi: 10.3390/nano9111636 (PMC6915603; doi:10.3390/nano9111636)
Supplement: Supplementary file 1 [file nanomaterials-09-01636-s001.pdf]

Supplementary Materials: One-Step Hydrothermal Synthesis of P25 @ Few Layered MoS_2 Nanosheets toward Enhanced Bi-catalytic Activities: Photocatalysis and Electrocatalysis

Fang Zhou [1,2,3], Zhiguang Zhang [3], Zhihua Wang [3], Yajing Wang [3], Liping Xu [3], Qiang Wang [2,*] and Wenjun Liu [1*]

[1] School of Physics, Harbin Institute of Technology, Harbin 150001, China; 15B911003@hit.edu.cn
[2] College of Physical Science & Technology, Yangzhou University, Yangzhou 225002, China
[3] College of Science & Information, Qingdao Agricultural University, Qingdao 266109, China; zhangzhiguang@qau.edu.cn (Z.Z.); misszhihua@qau.edu.cn (Z.W.); yajingphysics@qau.edu.cn (Y.W.); xlpzzd@qau.edu.cn (L.X.)
* Corresponding: wq@yzu.edu.cn (Q.W.); liuwenjun@hitwh.edu.cn (W.L.)

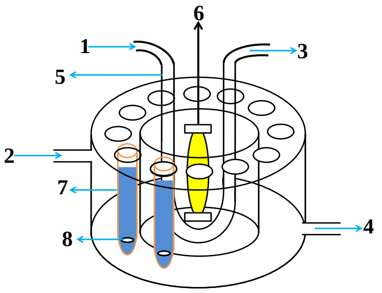

Figure S1. The schematic diagram of the photocatalytic system: (1) and (2) cooling water entrance, (3) and (4) cooling water outlet, (5) quartz well, (6) Xe lamp, (7) quartz reactor, (8) magnetic stirrer. The distance between the quartz reactor and lamp is about 12 cm, and the optical density of light source is about 120 mW/cm^2.

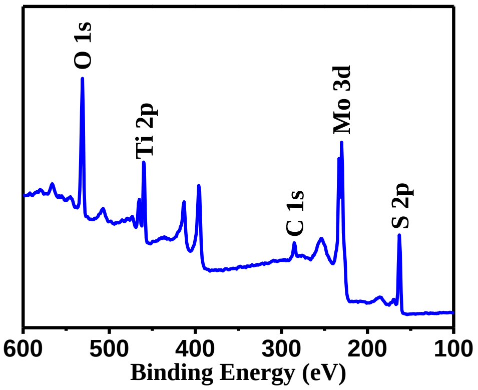

Figure S2. The full scan XPS spectrum of the PM11.

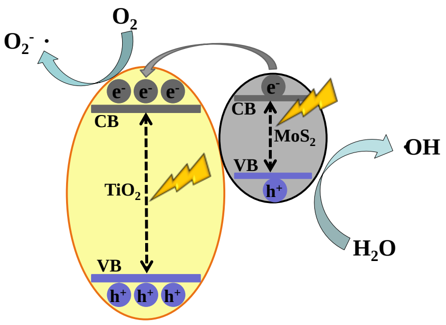

Figure S3. Schematic illustration of the mechanisms in degrading process.
